# Supplementary material for: Cross-Feeding of a Toxic Metabolite in a Synthetic Lignocellulose-Degrading Microbial Community
Source: Microorganisms. 2021 Feb 4;9(2):321. doi: 10.3390/microorganisms9020321 (PMC7914493; doi:10.3390/microorganisms9020321)
Supplement: Supplementary file 1 [file microorganisms-09-00321-s001.zip › microorganisms-1079004-S/lignocellulose_community_Figs_S1-S9_201230.pdf]

**Supplemental material for: Lee, J.; Baugh, A.; Shevalier, N.; Strand, B.; Stolyar, S.; Marx, J. Cross-feeding of a toxic metabolite in a synthetic lignocellulose-degrading microbial community.** ***Microorganisms*** **2021**.

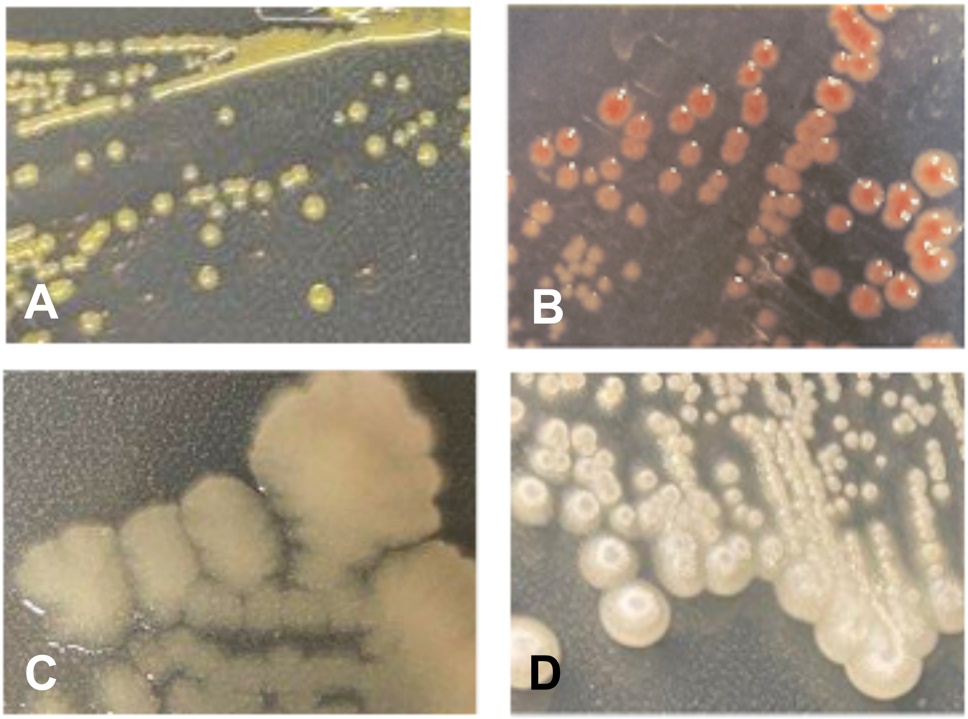


**Figure S1**. The four species in the consortium can be distinguished on agar plates by colony morphology. **(a)** *C. fimi* **(b)** *P. putida* **(c)** *M. extorquens* **(d)** *Y. lipolytica.*

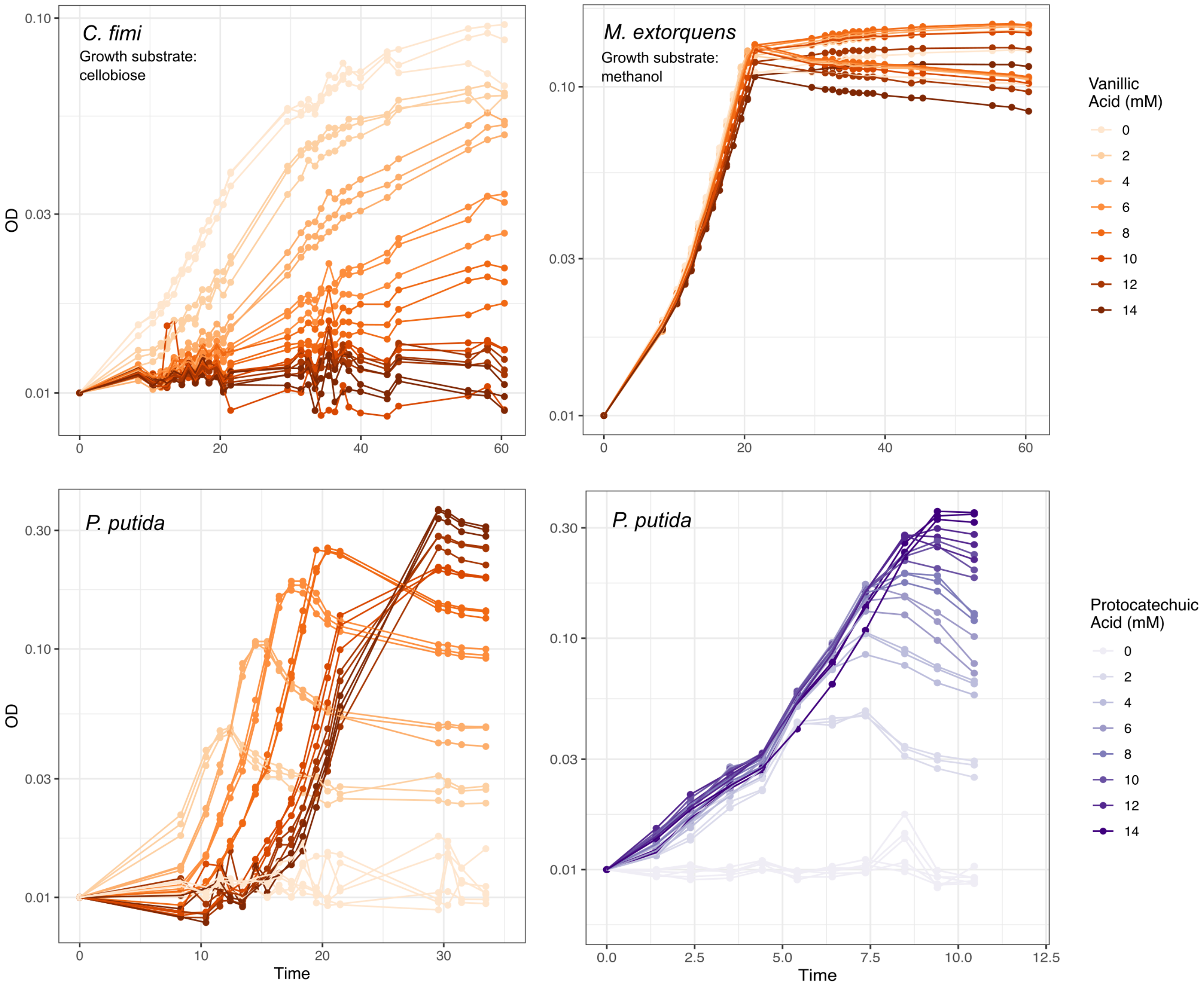


**Figure S2**. Growth curve data from growth rate experiments shown in Fig. 1

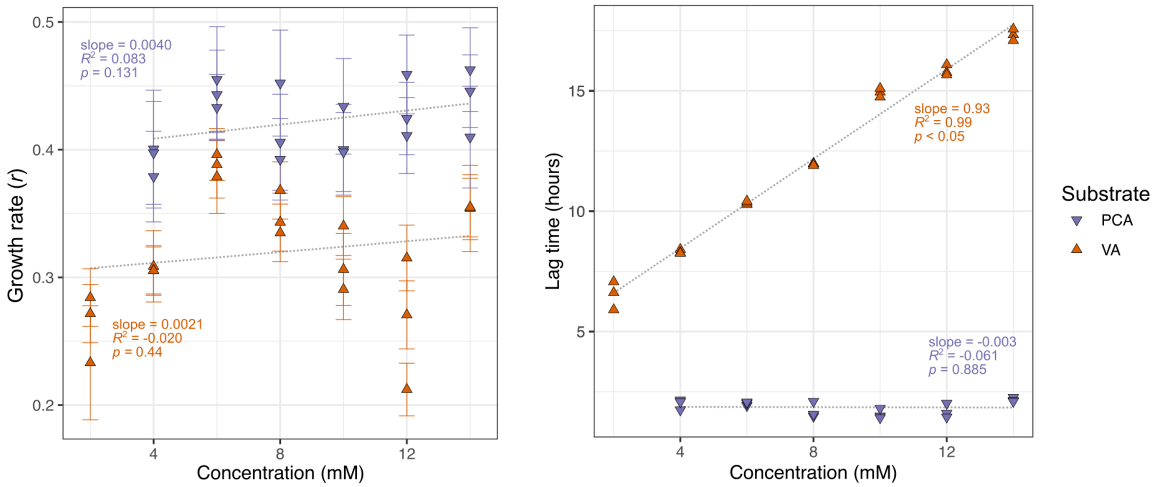


**Figure S3**. *P. putida* growth is inhibited more by vanillic acid than by PCA. *P. putida* was grown in minimal medium with either vanillic acid (orange triangles) or protocatechuic acid (inverted purple triangles) as a sole carbon source; growth was measured by $OD_{600}$. Only growth rates for which $R^2 > 0.9$ are shown here. At all concentrations, growth rates are slightly higher on protocatechuic acid than on vanillic acid. When growing on vanillic acid, lag time increases with concentration, while the same effect is not observed for protocatechuic acid.

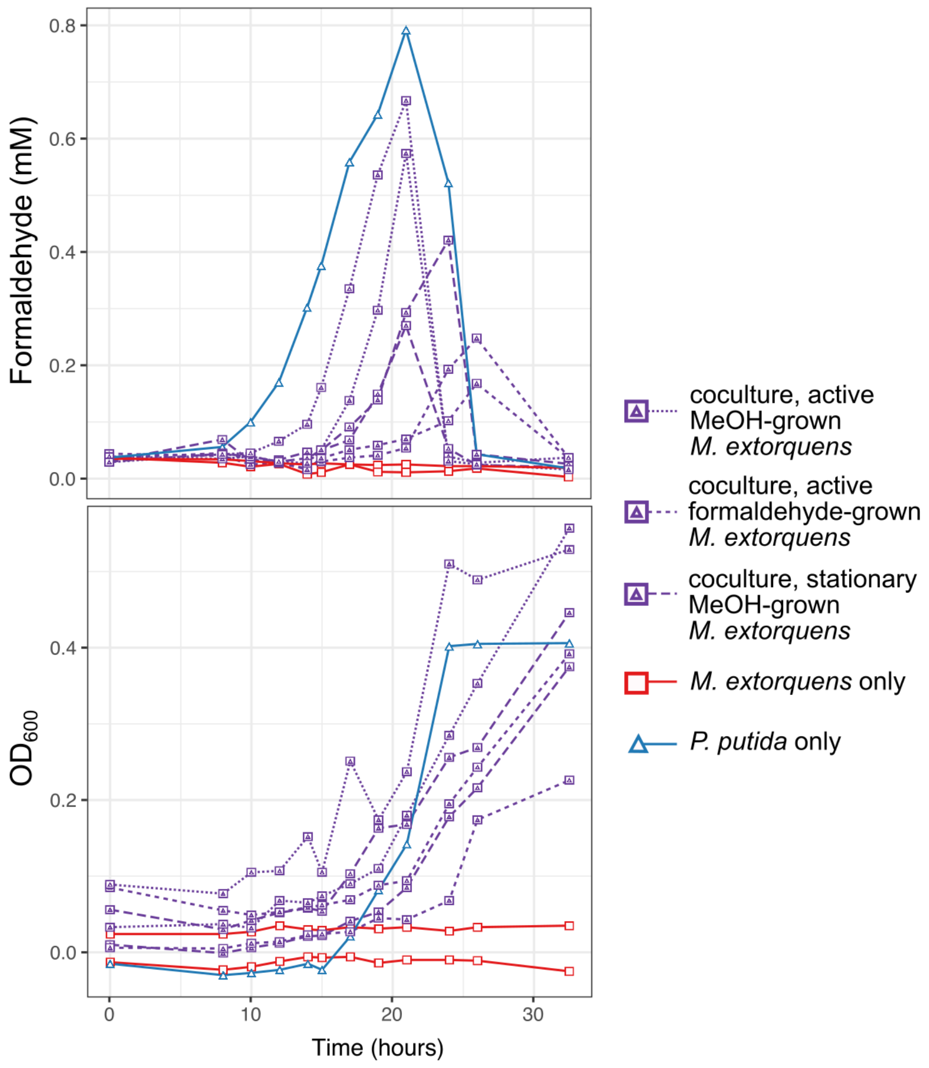


**Figure S4.** The conditions in which *M. extorquens* is grown prior to being added to the coculture influence how much formaldehyde it can consume, but all culture conditions lead to some formaldehyde reduction. The *M. extorquens* inoculum was pre-grown either on 15 mM methanol (MeOH) + 5 mM formaldehyde and inoculated at stationary phase (small-dash lines); or on 20 mM methanol alone and inoculated at either stationary phase (long-dash lines) or subcultured once and inoculated in exponential phase (dotted lines). *M. extorquens* inocula were normalized to the same OD prior to the experiment, and for each set of *M. extorquens* pre-growth conditions, two replicate experiments were conducted, with a twofold difference in the abundance of *M. extorquens* cells inoculated. Replicates are shown as separate lines in this plot. For all experiments, *P. putida* was inoculated in stationary phase. Pre-growing *M. extorquens* on formaldehyde substantially improved its ability to reduce the formaldehyde generated by *P. putida,* and actively-growing cultures were more effective than stationary-phase cultures.

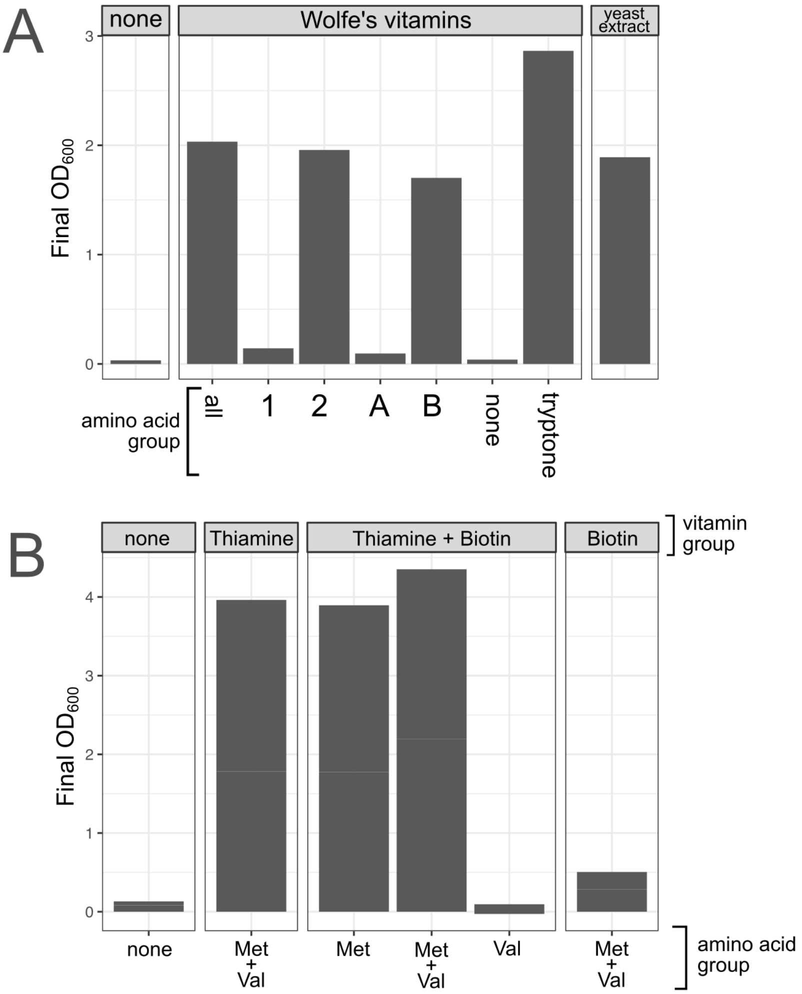


Experiment A amino acid groups

| Amino acid | Group letter | Group number |
|---|---|---|
| Alanine | A | 1 |
| Arginine | A | 2 |
| Asparagine | A | 1 |
| Aspartic acid | A | 2 |
| Glutamine | A | 1 |
| Glycine | A | 2 |
| Histidine | A | 1 |
| Isoleucine | A | 2 |
| Leucine | B | 1 |
| Methionine | B | 2 |
| Phenylalanine | B | 1 |
| Proline | B | 2 |
| Threonine | B | 1 |
| Tryptophan | B | 2 |
| Tyrosine | B | 1 |
| Valine | B | 2 |

**Figure S5**. Experiments in which *C. fimi* was grown on different groups of amino acids and vitamins were used to deduce its dependence on specific supplements for growth in minimal medium. **(a)** *C. fimi* was inoculated into MP minimal medium with glucose and either no vitamins, Wolfe's Vitamins, or yeast extract. Of the seven Wolfe's Vitamins cultures, each was supplied with a group of amino acids as denoted in the table, or with no amino acids ("none"), or with tryptone. *C. fimi* grew on amino acid group 2 and group B, allowing us to deduce that amino acid(s) required for growth were likely among the set methionine, proline, tryptophan, and valine. Further experiments were carried out testing these individually and in combination, as well as testing vitamins individually and in combination. **(b)** Final experiments indicated that methionine and thiamine were necessary and sufficient for growth by *C. fimi* on MP.

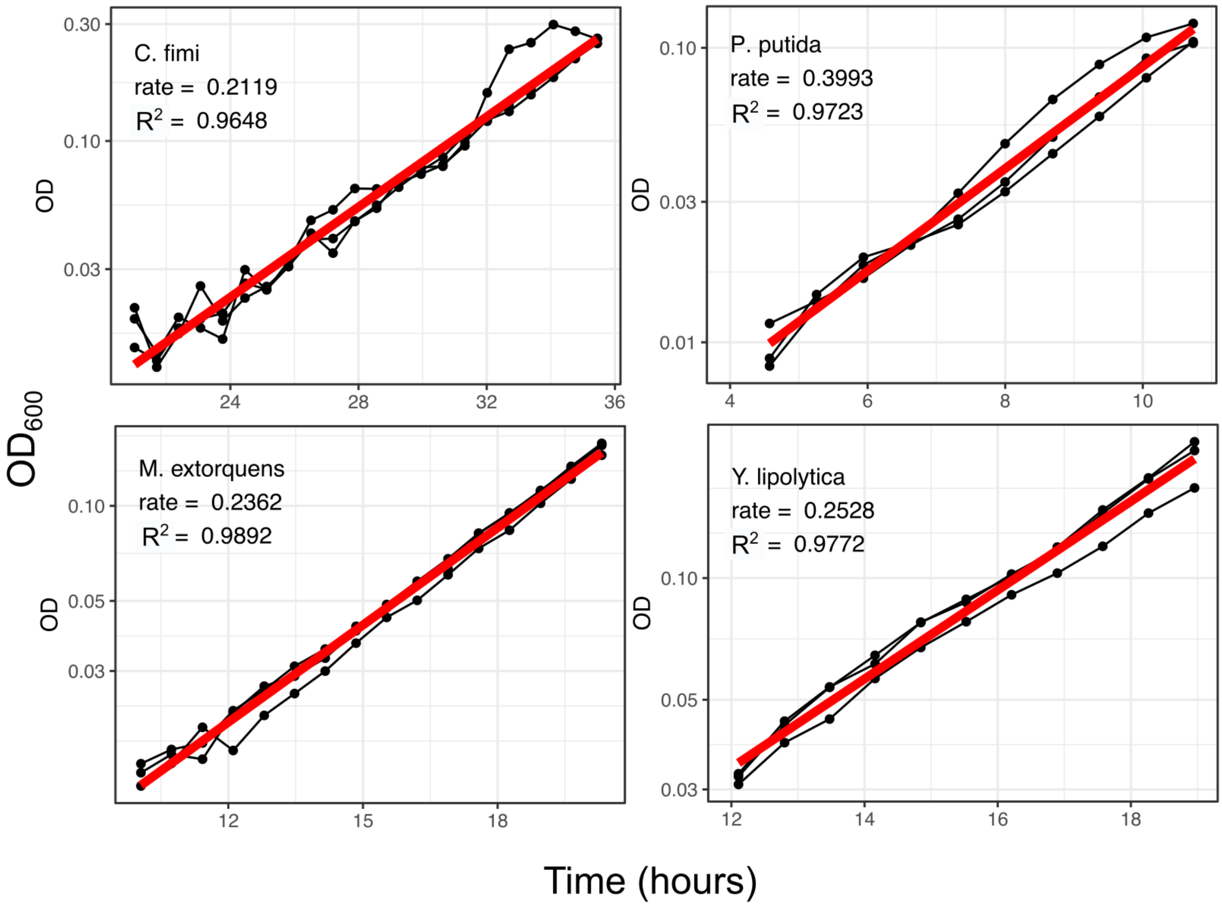


**Figure S6**. Growth rates of individual consortium members in optimal conditions. Each organism was grown in pure culture in MP medium on succinate (*M. extorquens*) or glucose (all other species) and methionine + thiamine + biotin supplements at standard concentrations. Growth was measured by OD. Only the points used for the calculation of growth rate, during exponential phase, are shown here. Replicate growth curves are connected by black lines. The red line shows the fitted rate.

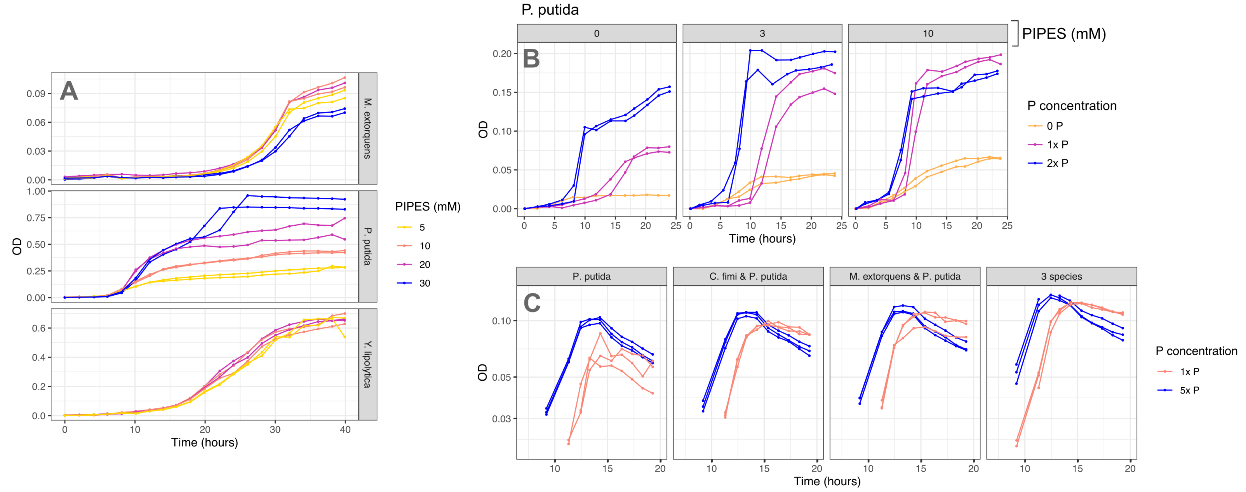


**Figure S7**. Of the consortium members, *P. putida* is the most sensitive to buffer concentrations. However, reducing the PIPES concentration to 0.1x can still support growth by *P. putida*, and increasing the phosphate concentration improves growth in low-PIPES medium. **(a)** Effect of PIPES concentration on each species, growing in MP medium with normal phosphate concentrations and methanol (*M. extorquens*) or glucose (other species). Note that OD is shown on a linear scale. Data are not available for *C. fimi*, or for *Y. lipolytica* at 30 mM. **(b)** Interaction of PIPES concentration (panels) and phosphate concentration (colors) on *P. putida* growing alone on glucose in MP medium. **(c)** Effect of phosphate concentration on coculture growth, growing in model lignocellulose medium with 0.1x PIPES. Standard concentrations in MP medium are 30 mM PIPES and approximately 3 mM phosphate; "5x P" therefore denotes approximately 15 mM phosphate.

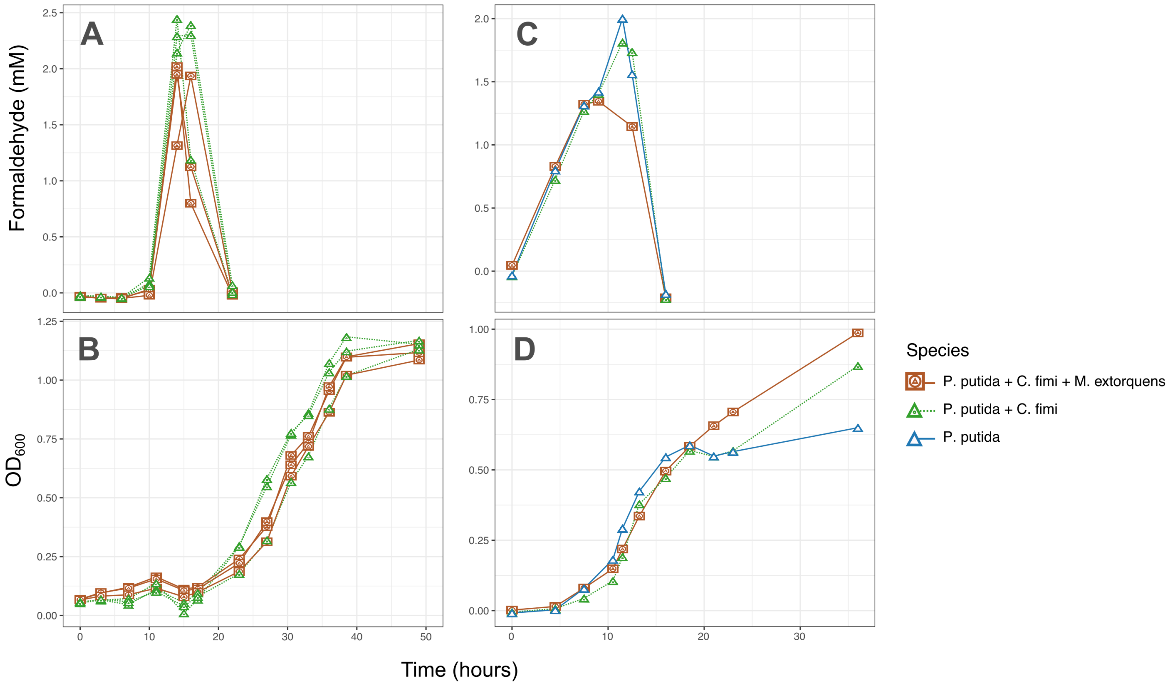


**Figure S8**. *M. extorquens* reduces formaldehyde accumulation but does not aid *C. fimi* growth in coculture in Model Lignocellulose medium. **(a-b)** *P. putida* and *C. fimi* were grown together either with or without *M. extorquens,* in Model Lignocellulose medium with an additional 10x Fe. Growth was measured by OD and cultures were sampled regularly for the measurement of formaldehyde in the medium. Each line denotes a replicate culture vessel. **(c-d)** similar to panels a-b, but medium was made with 1x PIPES, 1x phosphate, and 1x Fe, and measurements were also made on *P. putida* growing alone. In both cases, the formaldehyde peak is transient but there is at least one timepoint at which the culture with *M. extorquens* contains lower formaldehyde than the culture without, and the presence of *M. extorquens* does not have a significant effect on the growth curve.

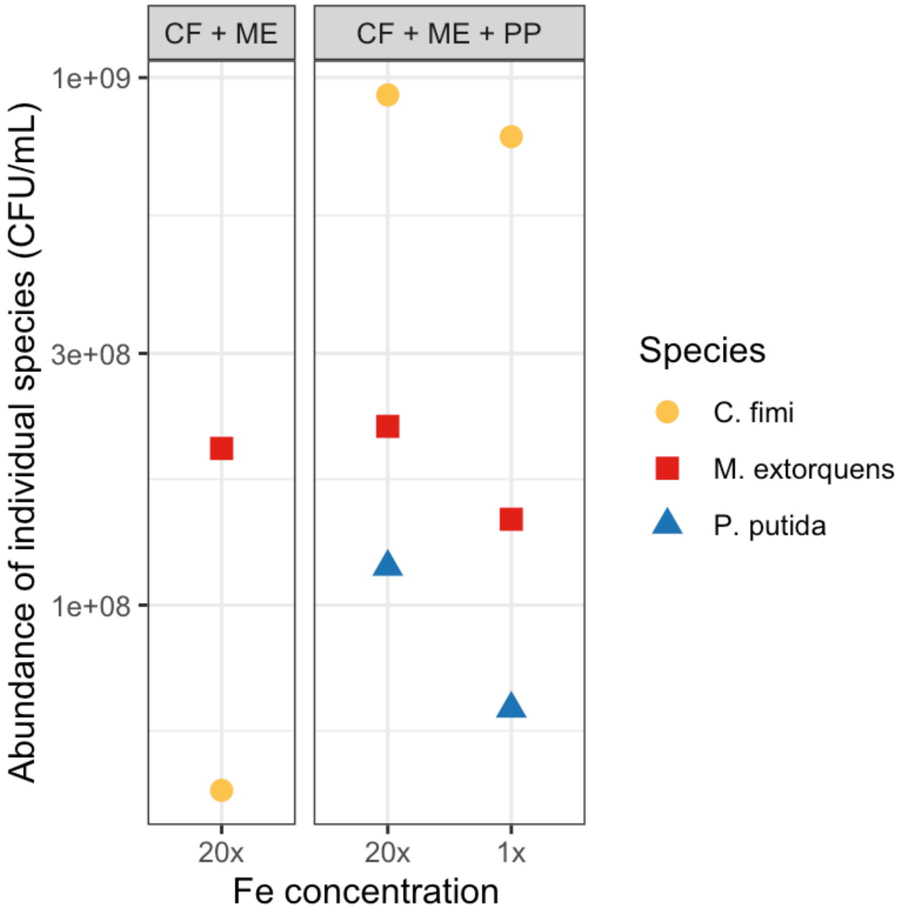


**Figure S9**. High concentrations of iron are inhibitory to *C. fimi* growth unless *P. putida* is present. *C. fimi* and *M. extorquens* were grown in Model Lignocellulose medium plus methanol, either with or without *P. putida* (panels) for approximately 48 hours, then plated onto agar medium. Species were identified by colony morphology. Note that the y-axis is on a log scale. When all three species are present, they reach higher abundance in the high-Fe medium. However, when *P. putida* is not present, *C. fimi* growth is dramatically impaired at high Fe concentrations. "1x Fe," the concentration typically in MP medium, is 17.8 μM.
